# Potential Harm of IQOS Smoke to Rat Liver

**DOI:** 10.3390/ijms241512462

**Published:** 2023-08-05

**Authors:** Silvia Granata, Donatella Canistro, Fabio Vivarelli, Camilla Morosini, Laura Rullo, Dario Mercatante, Maria Teresa Rodriguez-Estrada, Alessandra Baracca, Gianluca Sgarbi, Giancarlo Solaini, Severino Ghini, Ivan Fagiolino, Stefano Sangiorgi, Moreno Paolini

**Affiliations:** 1 Department of Pharmacy and Biotechnology, Alma Mater Studiorum, University of Bologna, Via Irnerio 48, 40126 Bologna, Italy; silvia.granata3@unibo.it (S.G.); donatella.canistro@unibo.it (D.C.); camilla.morosini2@unibo.it (C.M.); laura.rullo3@unibo.it (L.R.); severino.ghini@unibo.it (S.G.); stefano.sangiorgi9@unibo.it (S.S.); moreno.paolini@unibo.it (M.P.); 2 Department of Medicine and Surgery, University of Milan–Bicocca, Via Cadore 48, 20900 Monza, Italy; 3 Department of Agricultural and Food Sciences, Alma Mater Studiorum, University of Bologna, Viale Giuseppe Fanin, 40-50, 40127 Bologna, Italy; dario.mercatante2@unibo.it (D.M.); maria.rodriguez@unibo.it (M.T.R.-E.); 4 Inter-Departmental Centre for Agri-Food Industrial Research, Alma Mater Studiorum, University of Bologna, Via Quinto Bucci 336, 47521 Cesena, Italy; 5 Laboratory of Biochemistry and Mitochondrial Pathophysiology, Department of Biomedical and Neuromotor Sciences, University of Bologna, Via Irnerio, 48, 40126 Bologna, Italy; alessandra.baracca@unibo.it (A.B.); gianluca.sgarbi@unibo.it (G.S.); giancarlo.solaini@unibo.it (G.S.); 6 Gruppo CSA—S.p.A., Via al Torrente 22, 47923 Rimini, Italy; ifagiolino@csaricerche.com

**Keywords:** oxidative stress, liver disease, health risk, heated-tobacco products, drug metabolism

## Abstract

The Food and Drug Administration has recently classified the IQOS electronic cigarette as a modified-risk tobacco product. However, IQOS cigarettes still release various harmful constituents typical of conventional cigarettes (CCs), although the concentrations are markedly lower. Here, we investigated the damaging effects of IQOS smoking on the liver. Male Sprague Dawley rats were exposed, whole body, 5 days/week for 4 weeks to IQOS smoke (4 sticks/day), and hepatic xenobiotic metabolism, redox homeostasis and lipidomic profile were investigated. IQOS boosted reactive radicals and generated oxidative stress. Exposure decreased cellular reserves of total glutathione (GSH) but not GSH-dependent antioxidant enzymes. Catalase and xanthine oxidase were greater in the exposed group, as were various hepatic CYP-dependent monooxygenases (CYP2B1/2, CYP1A1, CYP2A1, CYP2E1-linked). Respiratory chain activity was unaltered, while the number of liver mitochondria was increased. IQOS exposure had an impact on the hepatic lipid profile. With regard to the expression of some MAP kinases commonly activated by CC smoking, IQOS increased the p-p38/p38 ratio, while erythroid nuclear transcription factor 2 (Nrf2) was negatively affected. Our data suggest that IQOS significantly impairs liver function, supporting the precautionary stance taken by the WHO toward the use of these devices, especially by young people and pregnant women.

## 1. Introduction

While tobacco consumption still remains one of the most urgent health emergencies of our time, with over 8 million deaths per year worldwide, new smoking devices have been introduced in the global market in the last decade. Nicotine delivery systems (NDSs) were basically designed to deliver smokeless nicotine from a propylene glycol/glycerol mixture (electronic NDS, ENDS, or electronic cigarettes, e-cigs) or by heating tobacco through a battery-powered heating system (heat-not-burn systems) such as the IQOS platform. Although e-cigs have been proposed as products with a lower health risk, there is no clear evidence to support the assertion that vaping is healthier than smoking, and there are no comprehensive studies to support the claim that ENDS can help smokers quit or reduce the health risk of smoking [1]. E-cigs can release irritant and carcinogenic compounds [2], in some cases even at higher concentrations than tobacco cigarettes [3], and in vivo analysis shows an overproduction of free radicals, which in turn leads to inflammation and a greater susceptibility to DNA damage, combined with the activation of co-carcinogenic pathways [4].

However, while the potential risks related to e-cig aerosol exposure are becoming clear, alternative devices equipped with new systems for delivering nicotine (such as IQOS) are not supported by adequate independent evidence of their possible toxicity. These heat-not-burn systems are extremely attractive to both smokers and never-smokers, especially young people, not least because the Food and Drug Administration (FDA) recently approved the marketing of IQOS as a modified-risk tobacco product (MRTP) [5], creating a sort of “illusion” about the product’s harmlessness. In reality, IQOS smoke contains carcinogenic by-products of pyrolysis and thermogenic degradation typical of traditional cigarettes, such as tobacco-specific nitrosamines, aldehydes, polycyclic aromatic hydrocarbons (PAHs), phthalates, carbon monoxide, volatile organic compounds and nicotine [6]. Moreover, some basic studies reveal not only that IQOS causes severe lung damage and promotes factors that advance the risk of lung cancer [7] but also that the product induces vascular cytotoxic outcomes comparable to those triggered by regular tobacco smoke [8].

It is interesting to note that IQOS smoking increases blood levels of alanine aminotransferase (ALT) as a marker of liver injury [9], suggesting that it could trigger unexpected organ toxicity not typically associated with tobacco smoke [10]. This study was, therefore, designed to explore the potential harmful effects of IQOS on carcinogen-metabolizing enzymes, redox homeostasis and lipid profile in rat liver by using an in vivo total-body exposure model.

## 2. Results

### 2.1. IQOS Does Not Affect Liver Weight

Animal weights were measured to assess their health, and organs were also weighed post mortem. Comparison of liver weights showed an absence of statistical differences, although a downward trend could be observed. Liver/body weight ratio analysis also revealed no differences between groups.

### 2.2. IQOS Increases Radical Oxygen Species (ROS) and Induces Oxidative Stress in the Liver

**Figure 1:**
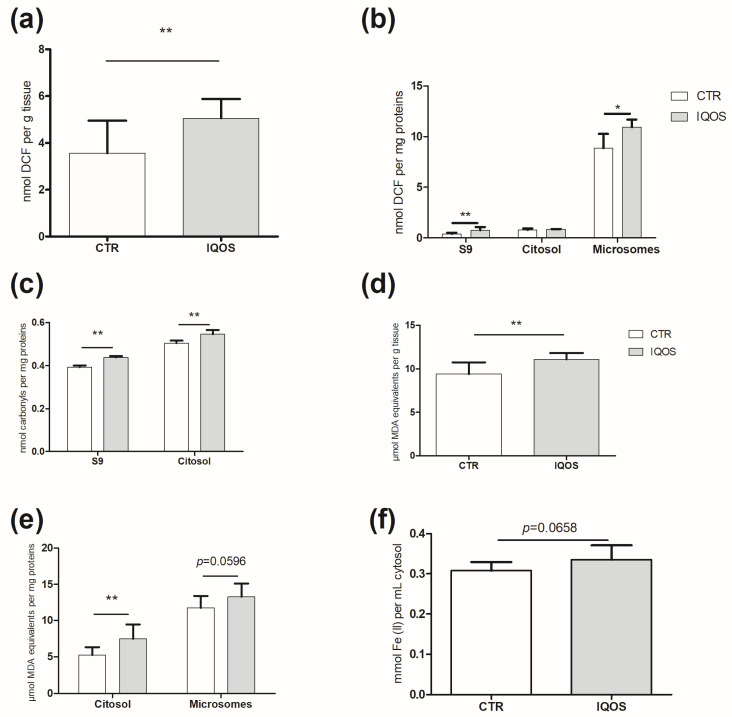
IQOS induces ROS and related damage in liver tissue. (**a**,**b**) ROS content in homogenate (**a**), S9, cytosol, and microsomes (**b**). Data are reported as nmol DCF per g of homogenized tissue and as nmol DCF per mg of proteins for other fractions. (**c**) CP in S9 and cytosol. Data are reported as nmol carbonyls per mg proteins. (**d**,**e**) MDA-equivalent concentration in homogenate (**d**), cytosol and microsomes (**e**). Data are reported as µmol MDA equivalents per g of homogenized tissue and as nmol MDA equivalents per mg of proteins for other fractions. (**f**) FRAP. Data are expressed as nmol Fe (II) per mL cytosol. *p* < 0.05 *, *p* < 0.01 **.

Dichlorodihydrofluorescein diacetate (DCFH-DA) assay revealed a statistically significant increase in ROS production in the treated group, in S9 and microsome fractions examined (*p* < 0.01, *p* < 0.05 respectively; Figure 1a,b). The greatest increase was detected in the S9 fraction, where samples from exposed animals showed almost a doubling compared to the control (*p* < 0.01; Figure 1b).

ROS overgeneration was associated with increased oxidative damage: a significant increase (*p* < 0.01) in carbonylated proteins (CP) in the exposed group, compared to the control group, was found in the S9 and cytosolic fractions (Figure 1c). Furthermore, the observed malondialdehyde (MDA)-equivalent concentrations increased in all of the fractions examined: a significant increase (*p* < 0.01) was detected in both the homogenate and cytosolic fraction (Figure 1d,e). Conversely, increased ROS did not cause significant growth in ferric-reducing/antioxidant power (Figure 1e).

### 2.3. GSH and Antioxidant Enzymatic System

**Figure 2:**
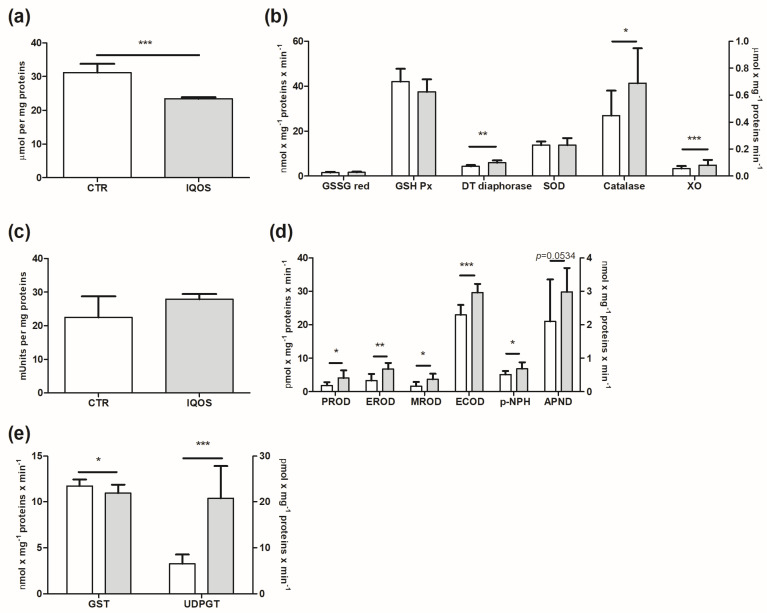
Total variations in GSH and antioxidant enzymatic systems, monooxygenases and phase II enzymes in rat liver following IQOS exposure. (**a**) Total amount of GSH. (**b**) GSSG red, GSH Px, DT−diaphorase, SOD (left y axis), catalase and XO (right y axis). (**c**) COX. (**d**) Phase I enzymes: PROD (CYP2B1/2), EROD (CYP1A1), MROD (CYP2A1), ECOD (not specific, CYP1A1, 1A2 and 2B) (left y axis); pNPH (CYP2E1) and APND (right y axis). (**e**) GST and UDPGT. Data are expressed as mean ± S.D. *p* < 0.05 *, *p* < 0.01 **, *p* < 0.001 ***.

Total glutathione (GSH) was strongly affected by IQOS smoking: the concentration of this substance in the treated group fell significantly (Figure 2a; *p* < 0.001). Antioxidant enzymes related to GSH, on the other hand, did not show significant changes. Levels of DT-diaphorase significantly increased in the exposed group compared to the control group (Figure 2b; *p* < 0.01).

Detoxifying enzymes were also affected: catalase increased significantly to approximately *p* < 0.05, while xanthine oxidase (XO) also showed a statistically relevant increase compared to the control group (Figure 2b; *p* < 0.001). 

### 2.4. Phase I Enzymes Are Upregulated by IQOS 

Monooxygenases were extensively evaluated, and all data showed an increase (Figure 2d). Pentoxyresorufin *O*-dealkylase (PROD), ethoxyresorufin *O*-deethylase (EROD) and methoxyresorufin *O*-demethylase (MROD) were the oxidases showing the highest increases. In addition, p-nitrophenol hydroxylase (p-NPH) and ethoxycoumarin *O*-deethylase (ECOD)-linked oxidases displayed a lower but statistically significant rise. It should be noted that, although the increases in p-NPH and ECOD were apparently less than that of monooxygenases probed by resorufin, the fact that these oxidases are expressed in nmol (mg × min)^−1^ compared to others based on resorufin that are expressed as pmol (mg × min)^−1^ means that the corresponding absolute increases should be considered.

### 2.5. Phase II Enzymes

Glutathione S-transferase (GST) and the uridine diphosphate-glucuronosyltransferase (UDPGT)-dependent activities considered to be phase II markers showed opposing trends: GST was reduced (*p* < 0.05) in the exposed group, while UDPGT increased by three times as much compared to the control group (Figure 2e; *p* < 0.001).

### 2.6. IQOS Affects Mitogen-Activated Protein Kinase (MAPK) p38 and Erythroid Nuclear Transcription Factor 2 (Nrf2)

**Figure 3:**
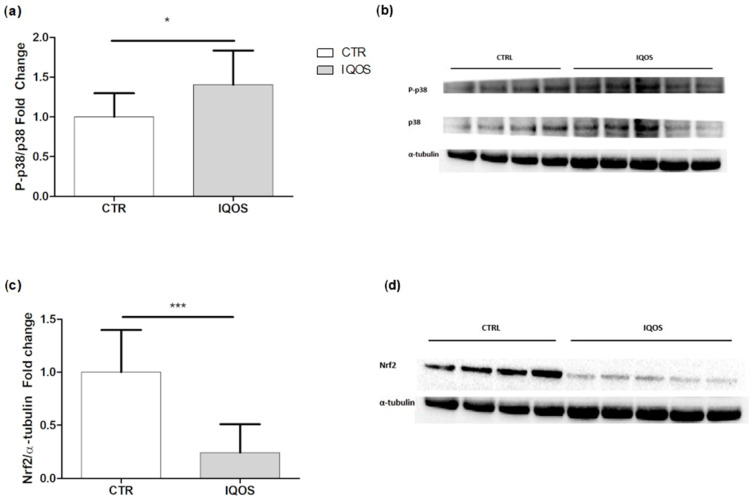
Western blot analyses of MAPK p38 and Nrf2 expression in liver tissue. (**a**) p-39 MAPK expression: bars represent the mean ± SD of the phosphorylated form/total protein ratio, and α- tubulin is used as a loading control. (**b**) Representative image of the blot. (**c**) Nrf2 expression: bars represent the mean ± SD of the phosphorylated form/total protein ratio, and α- tubulin is used as a loading control. (**d**) Representative image of the blot. *p* < 0.05 *, *p* < 0.001 ***.

Western blot analysis for both markers showed opposing trends: the phospho-p38/p38 ratio showed a statistically significant increase in the treated group compared to the control group (Figure 3a), whereas Nrf2 was more than halved (Figure 3c).

### 2.7. Respiratory Chain Activity and Mitochondrial Content

Complex II-driven respiratory chain activity was evaluated under uncoupling condition in isolated liver mitochondria. The maximum oxygen consumption rate, measured in the presence of the uncoupler carbonyl cyanide 4-(trifluoromethoxy)phenylhydrazone (FCCP), showed a slight but non-significant increase in mitochondria of treated rats compared to controls (Figure S1A,B). Due to possible differences in the mitochondrial content of each preparation, we assessed citrate synthase (CS), a mitochondrial matrix enzyme widely considered an index of mitochondrial mass. CS showed a slight but non-significant increase in IQOS-exposed livers compared to controls (Figure S1C). Consequently, the normalization of the oxygen consumption rate to CS activity showed no changes between the experimental groups considered (Figure S1D).

To evaluate the impact of the main mitochondrial functions on cell metabolism, the content of mitochondria was assessed. Total CS activity present in the crude extracts was assayed, and the activity was then normalized to the weight of the liver before its rupture. This made it possible to quantify the mitochondrial content per gram of liver. There was a significant increase (*p* < 0.05) in mitochondrial mass in the treated samples compared to controls (Figure S1E).

### 2.8. IQOS Alters Lipid Profile

**Figure 4:**
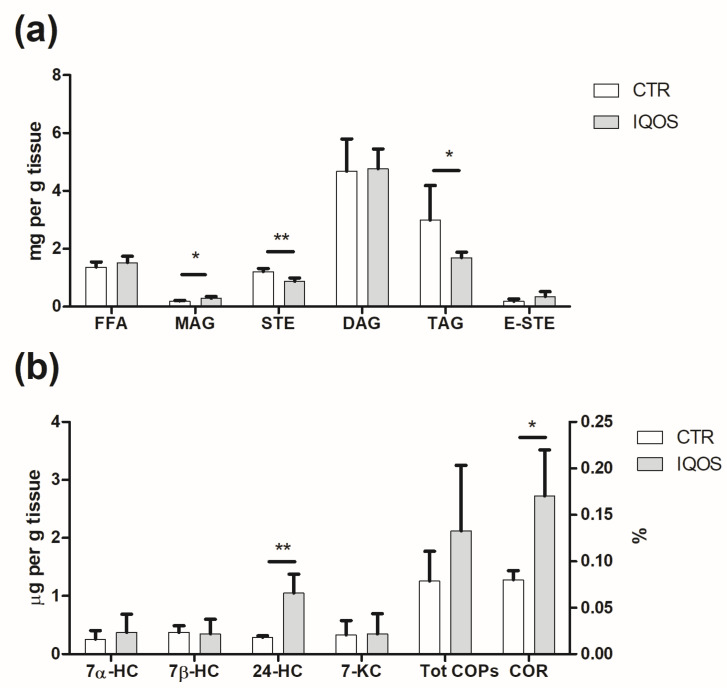
Lipidomic analysis of the liver. (**a**) MAG increased in the treated group compared to the control group; both STE and TAG decreased (**b**), while only 24-HC and COR% showed an increase. *p* < 0.05 *, *p* < 0.01 **.

Overall lipid composition of rat liver (Figure 4a) was markedly affected by IQOS, showing a decrease in triacylglycerol (TAG) and free sterol (STE) (*p* < 0.01 and *p* < 0.05, respectively) and an increase in monoacylglycerol (MAG) in treated livers (*p* < 0.05). No significant differences were observed in free fatty acids (FFA), diacylglycerols (DAG) and esterified sterols (E-STE).

In terms of fatty acid (FA) composition (Table S2), the most commonly represented FA class was saturated FA (SFA), followed by polyunsaturated FA (PUFA) and monounsaturated FA (MUFA). The most abundant FAs were palmitic (C16:0), linoleic (C18:2) and oleic acids (C18:1). No significant effect of IQOS treatment was detected on individual FA, the main FA classes, their ratios and the Δ-desaturase index (Table S2). A non-significant, increasing trend in MUFA, total PUFA, n-3 PUFA, n-6 PUFA, their ratios (n-6/n-3, PUFA/SFA, UFA/SFA) and the Δ-desaturase index was observed in the livers of IQOS-exposed rats.

Based on FA composition, AI and the thrombogenic index (TI) were also calculated, as these values are useful indices in understanding the role of FA composition in both atherogenic and thrombogenic risks: both AI and TI were slightly increased (Table S2).

Regarding total cholesterol content, no significant differences were found between controls (1.57 ± 0.40 mg g^−1^ of liver) and treated livers (1.27 ± 0.47 mg g^−1^ of liver), although a decreasing trend could be seen in those exposed to IQOS. In terms of cholesterol oxidation (Figure 4b), the total oxysterol level was 1.26 and 2.12 µg/g of liver in the controls and the IQOS-exposed samples, respectively, demonstrating the pro-oxidant effect of this device. Oxysterols found in both samples were 24-hydroxycholesterol (24-HC), 7α-hydroxycholesterol (7α-HC), 7β-hydroxycholesterol (7β-HC) and 7-ketocholesterol (7-KC). Significant differences were found only in 24-HC (*p* < 0.01 and *p* < 0.05), with values 3.6 times higher in liver from IQOS-treated animals compared to controls. The doubling of the cholesterol oxidation ratio (COR%, Figure 4b) confirmed the pro-oxidant effect of IQOS on the liver (*p* < 0.05).

### 2.9. Principal Component Analysis (PCA) Reveals IQOS Impact on Liver Function

To better assess the impact of IQOS smoking on the rat liver and the data variability and the interrelationships between different variables using the data projection method, selected results were subjected to PCA. The first two principal components reached a variance of 60.64%, and PC1 and PC2 explained 31.97% and 28.67% of that variance, respectively. In quadrant 2 (Figure S2), it is possible to observe a cluster of variables related to oxidation (TBA cytosol, 24HC, Xantine Ox, COR%, MROD, UDPGT, PROD, EROD, CP cytosol, catalase, desaturase, ECOD, ROS S9, pNPH), which mostly contribute to PC1, with values between 2 and 8% and that were closely related to each other, forming an angle of amplitude of less than 90°. These variables were also closely related to the treated (T) group and inversely correlated with the control (C) group. The two experimental groups, in fact, were well separated from each other, being mostly concentrated in two different areas of the biplot. As shown in Figure S2, the C group was characterized mainly by STE, GSH Tot, SOD, total cholesterol, TAG and STE, and was inversely correlated to the aforementioned oxidative parameters and some lipolysis indices (MAG, FFA), which, in turn, reflected the T group.

## 3. Discussion

The FDA recently labeled IQOS as an MRTP, suggesting a misleading perception of a “clean” and safe product among consumers [5]: IQOS smoke contains a concentration of toxic substances lower than that in conventional cigarette (CC) smoke [11], but still contains many potent carcinogens, some at the same levels (such as formaldehyde) or even three times higher (such as acenaphthylene) than in cigarettes. JAMA editor Mitchell H. Katz [12] said these devices in fact release classic carcinogens, even though some scholars argue about the “smoke” label for the emissions generated by IQOS, further suggesting that existing smoking bans should be amended to include these products. The WHO emphasizes that there is no clear threshold value for the toxic effects induced by the “low level of carcinogens” in second-hand smoke, although we should not forget that indoor (IQOS) smoking causes an accumulation of toxins. The smoke generated by the IQOS tobacco sticks used in our study, as reported in our publication on the lung [7], contains IARC Class 1 human carcinogens such as aldehydes (for example, acetaldehyde and formaldehyde), PAHs, benzene, volatile organic compounds and nicotine (Table S1) [7]. A total switch from CC to IQOS appears to be associated with a reduction of some harmful effects [13], but more evidence is needed to evaluate the overall health risks for never-smokers and, especially, more vulnerable young people.

We found that IQOS induces oxidative stress, which is known to cause various liver pathologies such as steatosis and cirrhosis and alters the physiological functions of the liver, thus contributing to the development of these diseases [14]. Our finding is also worrying because a pathological inflammatory response could be promoted by elevated ROS levels [15], as they were in our study. Curiously, the concentration of ROS present among the constituents of IQOS smoke was similar to the urban concentration of ROS [11].

We found a significant induction of various monooxygenases and XO, along with an enhancement in mitochondrial tissue respiration. Analyzed CYPs were boosted by IQOS, thereby increasing individual toxicological risk due to the increased bioactivation of both IQOS smoke constituents and ubiquitous pre-mutagenic chemicals [16]. IQOS consumers will in fact be exposed not only to the carcinogens produced by the tobacco stick, which in themselves do not have a threshold for damaging effects on DNA, but also to the omnipresent carcinogens with which consumers come into daily contact (air pollution, drinking water, food chain), which unfortunately have a cumulative effect. Thus, increased bioactivation by CYP1A1/2 isoforms of aromatic amines, dioxins and PAHs, as well as increased bioactivation by CYP2B1/2 isoforms of olefins and halogenated hydrocarbons, for example, could overload the cell’s DNA repair enzyme system, thereby promoting carcinogenesis [17]. Stimulation of CYPs has also been linked to an overproduction of superoxide anion, released from the same catalytic cycle as P450 for the dissociation of the ferrous-dioxygen complex, generating further oxidative stress that can alter immune response and cause non-alcoholic fatty liver disease (NAFLD) [16,18]. Overall, any change in CYPs, which is already complicated by differences in personal metabolism due to polymorphisms, can only increase an individual’s toxicological risk [19]. Just as cigarette smoking manipulates the CYP superfamily by altering drug bioavailability [20], IQOS modifies pharmacokinetics, making drug effects unpredictable.

While an increase in liver weight after 90 days of IQOS treatment was reported as a sign of hepatocellular hypertrophy [21], no differences were observed under our conditions, possibly because of the shorter period of exposure. Conversely, data obtained from mitochondrial analysis show that livers increased their mitochondrial mass, most likely through activation of the biogenesis process to counteract possible damage to oxidative phosphorylation caused by ROS.

Despite the fact that cellular mechanisms usually respond to ROS overgeneration with an increase in antioxidant defenses, we did not observe any major changes in the antioxidant apparatus, with the exception of a boost in DT diaphorase and catalase. FRAP, which was also assessed as an index of antioxidant power [22], was slightly affected. Nonetheless, consistently with CC smoking studies, we found a significant reduction in total GSH, probably caused by radical stress: in patients with chronic liver disease, GSH has been observed to decrease significantly [23], and changes in the homeostasis of the GSH system play a role in the etiology and development of liver disease, including cancer [24]. Regarding post-oxidative defenses, we observed an increase in UDPGT in line with that caused by CC smoking in the liver and in other organs after exposure to ENDS, and upregulation of this substance occurs in patients with cirrhosis [25]. Conversely, GST was lower in the exposed group. Thus, IQOS exposure might be detrimental to liver health.

We found a significant reduction in Nrf2 expression in the liver of IQOS-exposed rats, which is in agreement with evidence that chronic smoke exposure from CC can impair activation of this process [26]. Here, we observed that reduced Nrf2 expression was associated with an increase in p38. Indeed, MAPKs are engaged in the regulation of Nrf2: it is believed that Nrf2 is downregulated by activation of p38, while it is promoted by activation of JNK1 and ERK2 [27]. It has been suggested that this modulation plays a key role in inducing liver damage caused by tobacco smoke, regardless of its combustion process [28]. Nrf2 reduction combined with p38 activation are clear signs that IQOS exposure results in an impairment in liver function in the same way as tobacco smoke, which is known to induce MAPK activation and lung cancer [29].

Oxidative stress can also promote protein carbonylation, one of the irreversible oxidative protein alterations and a major hallmark of oxidative damage, which has a role in cell signaling and toxicity and is involved in inflammation, autoimmune responses, cholestatic liver disease, primary sclerosing cholangitis (PSC), NASH, Alzheimer’s disease, muscular dystrophy, atherosclerosis and respiratory syndrome [30]. Increased carbonylation was clearly found in our investigation. We showed a significant increase in lipid peroxidation, which is known to be related to loss of membrane function, decreased fluidity, DNA and protein damage, disruption of gene functions and promotion of cell death pathways, such as apoptosis and necrosis, and can produce liver inflammation and fibrosis by activating the stellate cell compartment, eventually leading to organ failure [31].

Lipidomic analysis provided evidence of a lipolytic process, particularly evident in TAGs, while MAGs showed a marked increase. A growing trend was also observed for FFA and DAG, which was additional confirmation of the occurrence of lipid hydrolysis. With regard to PUFA, an increasing trend was observed in the Δ-desaturase index: both Δ5- and Δ6-desaturases are involved in the formation of n-6 and n-3 long-chain PUFA (LCPUFA), with Δ-6 desaturase being the limiting enzyme in the process. Desaturases are regulated by hormones such as insulin and estrogens and by the intracellular redox state, and are subjected to polymorphisms, resulting in alteration of tissue n-6 and n-3 LCPUFA levels [32]. Oxysterol analysis confirmed the induction of oxidative stress by IQOS smoking, with the level of 24-HC in the treated livers being higher than in the controls. Oxysterols play a critical role in many regulatory processes: they activate ligands of liver X receptor and are involved in various diseases, such as fat-induced injury and liver injury in non-alcoholic fatty liver disease [33]. Oxysterols (mainly 7α-HC and 27-HC) are also intermediaries in bile acid synthesis and are involved in cholesterol homeostasis through suppression of LDL receptors and 3-hydroxy-3-methylglutaryl coenzyme A (HMG-CoA) reductase. Here, most COPs displayed similar behavior, except for 24-HC, which was significantly increased by IQOS. 24-HC is generated by 24-hydroxylase (CYP46A1) and is largely expressed in neurons and neural retina. It is important to note that an increased accumulation of 24-HC at the neuronal level leads to neurodegenerative disorders such as Alzheimer’s disease, Huntington’s disease, Parkinson’s disease, demyelinating diseases and multiple sclerosis [34]. Further studies are needed to better clarify this phenomenon.

The multivariate data analysis by PCA, related to lipid peroxidation, protein oxidation and glycoxidation, confirmed that IQOS exposure has an impact on rat liver, suggesting that these phenomena should be investigated thoroughly, especially in humans.

To our knowledge, this is one of the emerging toxicological in vivo rat liver studies on IQOS. Overall, we showed that the IQOS device is harmful not only to primary target organs such as the lungs but also to the liver.

The present work represents one of the few independent studies on the toxicological effects of unburned-tobacco devices such as IQOS in the liver as a secondary target organ in an in vivo model. It should not be interpreted as a translational study, and the results presented cannot be directly extrapolated to humans. We discuss the potential effects of smoking IQOS on the hepatic P450 system resulting in increased bioactivation capacity, increased reactive oxygen species generating oxidative stress, along with impaired lipid metabolism. The present study lacked a histopathological evaluation, and it did not take into account blood markers of liver damage. These data could have provided precious information on the health status of the animals. The study was conceived as an exploration of the toxicological effects of IQOS per se and would benefit from a comparison with a positive control group exposed to tobacco cigarettes. Finally, the study was carried out without the use of a professional nose-only exposure system, and puff topography did not address ISO standards as well as other aerosol characterization measures recently reported by Noel and colleagues [35]. However, our total-body model of exposure limited the stress of restraint required for nose-only exposure and the alteration of respiratory rate/volumes/flows that can impact toxicological outcomes. Independent studies on the toxicological effects associated with IQOS consumption are still scanty, and we believe that our work, being the first in vivo study on the liver, may be of interest despite its limitations for the scientific community in laying the foundation for future investigations.

## 4. Materials and Methods

### 4.1. Chemical Analysis of IQOS Mainstream Aerosol

Chemical analysis of IQOS mainstream aerosol was performed by using GC/MS. The full list of compounds analyzed in this study and the methods used are reported in Table S1.

### 4.2. Animal Exposure

The experiments were carried out in accordance with the EU Directive 2010/63/EU. The protocol received approval from the Ethics Committee for Animal Experiments of the University of Bologna and the Ministry of Health (Permit number: 2683015). Twenty-four male Sprague Dawley rats (ENVIGO RMS S.r.l., San Pietro al Natisone, Udine, Italy), 7 weeks old, were housed under standard conditions (12 h light/dark cycle, 22 °C, 60% humidity). After 2 weeks of acclimatization, animals were randomly assigned to the control (n = 14 rats) or exposed (n = 10 rats) group. Exposure settings were comparable to those described in previous studies [7]. Control animals were exposed to air but subjected to the same procedure of manipulation as the IQOS group. The IQOS group was subjected to total-body exposure. The inhalation chamber consisted of a propylene box (38 cm × 26.5 cm × 19 cm) with a capacity of 19 L. The pump (0.18 kW; 1.4/1.6 A; 230 V; 50/60 Hz) was installed on one side of the box, while the IQOS aerosol was puffed on the other, generating the airflow into the chamber. The chamber, containing 2 animals at a time, was not hermetically sealed, and the 3 holes (2 IQOS and pump connection points) were never occluded during the experimental procedure. The puff profile (5 s on, 15 s off, 5 s on) with an air flow of 4 L/min was set as previously described [36]. Two IQOS were connected in tandem and, therefore, the flow rate generated by the pump was applied and distributed to both devices. The exposure session ended when the IQOS stick was consumed and the device automatically turned off; rats were then held up for 20 min. Animals were exposed for 5 consecutive days/week to a total of 904 µg nicotine/chamber/day. The experiment was prolonged for 4 weeks, and the Animal Welfare Committee monitored the animals throughout the entire experiment. The concentration of nicotine recorded in IQOS smoke was 113 ± 26 µg/stick [7], which is significantly lower than the LC50 for vaporized nicotine in the rat (2.3 mg/L) [37].

### 4.3. Tissue Collection and Sub-Cellular Fraction Isolation

Rats were sacrificed following the Italian Ministerial guidelines for the species, and the fractions were isolated as previously reported [38]. Liver mitochondria were isolated in accordance with Barogi et al. [39]. See Supplementary Material for detailed information.

### 4.4. Protein Concentration

The protein concentration of the collected fractions was determined by the method described by Lowry et al. and modified as previously reported [40].

### 4.5. DCHF-DA Assay for Reactive Oxygen Species (ROS) Estimation in Tissue Homogenate and Subcellular Fractions

ROS were evaluated throughout the 2′,7′-dichlorofluorescein diacetate (DCFH-DA) assay [41]. 2′,7′-Dichlorofluorescein diacetate (DCFH-DA) was used as probe for the estimation of ROS content in tissue homogenate. Samples were mixed with DCFH-DA (100 μM) at 37 °C for 30 min, and the reaction was shut down by chilling. The formation of the oxidized breakdown product 2′,7′-dichlorofluorescein (DCF) was monitored by means of a fluorescence spectrophotometer (EX = 488 excitation; EM = 525 emission). A solution of dichlorofluorescein was prepared at different concentrations and used for the calibration curve.

### 4.6. Ferric Reducing Antioxidant Power (FRAP)

The assay was performed on the cytosolic fraction [42]; for the calibration curve, scalar concentrations of ferrous sulphate solution were used; samples were read at λ 593 nm.

### 4.7. Thiobarbituric Acid Reactive Substance (TBARs)

The malondialdehyde (MDA)-equivalent concentration was determined in homogenate, S9, cytosol and microsomal fraction by using a wavelength at λ = 532 nm [43].

### 4.8. Carbonylated Proteins (CP)

Briefly, samples were mixed with 2,4-dinitrophenylhydrazine solution 10 nM, then with trichloroacetic acid solution 20% (TCA), and centrifuged at 4000 rpm for 10 min at 4 °C. The pellet was resuspended and again centrifugated at the same conditions; the supernatant obtained was read at λ = 390 nm [44].

### 4.9. Antioxidants

Total glutathione (GSH) assay, GSH peroxidase (GSH Px), glutathione disulphide reductase (GSSG Red), DT-Diaphorase, catalase (CAT), superoxide dismutase (SOD) and xanthine oxidase (XO) was performed as described in Cirillo et al. [2]. See Supplementary Material for detailed information.

### 4.10. Cyclooxygenase (COX)

The microsomal fraction was mixed with Tris-HCl 100 mM EDTA 3 μM solution pH = 8.0 and N,N,N’,N’-tetramethyl-p-phenylenediamine solution 133 μM. The reaction was activated through the addition of arachidonic acid and followed at λ = 603 nm from t0 to t1 (1 min) [S11].

### 4.11. Phase I Enzymes

CYP-linked monooxygenases p-Nitrophenol hydroxylase (p-NPH, CYP2E1), pentoxyresorufin *O*-dealkylase (PROD, CYP2B1/2), ethoxyresorufin *O*-deethylase (EROD, CYP 1A1) and methoxyresorufin *O*-demethylase (MROD, CYP1A2), ethoxycoumarin *O*-deethylase (ECOD, CYP1A1, 1A2 and 2B), and aminopyrine *N*-demethylase (APND, CYP3A1/2) were assessed as previously reported [45]. Detailed procedures are described in the Supplementary Material.

### 4.12. Phase II Enzymes

UDP-glucuronosyl transferase (UDPGT) was measured in the microsomal fraction using 1-naphtol as substrate by the fluorometric recording of 1-naphtholglucuronide production in the presence of uridine-5′-diphosphoglucuronic acid. EX = 293 nm, EM = 335 nm and slit = 5/5 [46]. Glutathione S-transferase (GST) was determined as previously described [38]. The incubation mixture contained 0.1 M phosphate Na^+^/K^+^ buffer (pH 6.5), 1 mM glutathione (GSH) and 1 mM 1-chloro-2,4-dinitrobenzene (CDNB) dissolved in methanol. Once cytosol was added, the product of the reaction of the thiol group of GSH with the electrophilic group of CDNB was followed at 340 nm (ɛ = 9.6 mM^−1^ cm^−1^). UDP-GT was determined in microsomal fractions All details have been reported previously.

### 4.13. Western Blot

Lung protein extraction was performed employing the T-PER Tissue protein extraction reagent (Thermo Scientific, Waltham, MA, USA) and Halt Protease and Phosphate inhibitor cocktail (Thermo Scientific) following the manufacturer’s recommendation. Protein quantification was performed using the Pierce BCA Protein Assay Kit following the guidelines. Proteins were separated in one dimension on Bolt 4–12% Bis-tris Plus gels (Invitrogen Thermo Scientific, Waltham, MA, USA) using a mini protean vertical gel electrophoresis mini-tank module (Invitrogen Thermo Scientific). The detailed procedure is included in the Supplementary Material section. See Supplementary Material for more information.

### 4.14. Respiration Rate Measurement

Oxygen consumption rates were measured at 37 °C using a Clark-type oxygen electrode as previously reported [47].

### 4.15. Citrate Synthase and Protein Assay

Citrate synthase (CS) was detected by incubating 10–50 μg mitochondrial protein in 1 mL of 0.125 M Tris–HCl, 0.2% Triton X-100, 0.1 mM acetyl-coenzyme A, 0.1 mM 5,5′-dithio-bis(2-nitrobenzoic acid) (DTNB), and 0.5 mM oxaloacetate. Activity was assessed by monitoring at 412 nm the release of 2-nitro-5-thiobenzoate (ε = 13.6 mM^−1^ cm^−1^) [48].

### 4.16. Lipid Analysis

Lipids were extracted as described by Boselli et al. [49] Profiles of the main lipid classes were determined and further analyses were performed as described in the Supplementary Material.

### 4.17. Statistical Analysis

Unless otherwise specified, data are presented as mean ± SD. Datasets from enzymatic assays, oxidative stress markers (such as the TBARs assay, lipid peroxides, protein carbonyl groups) and immunoblotting were tested for normality by the D’Agostino and Pearson or Kolmogorov–Smirnov test and analyzed using the two-tailed unpaired *t*-test or Mann–Whitney test in the case of non-normality distribution. Results for mitochondrial uncoupled respiration were statistically analyzed by comparing the treated animals vs. the control group by means of a paired Student’s *t*-test. Statistical significance was set at *p* < 0.05. Results from the lipidomic analysis are reported as mean ± SD of eight animals (n = 8) for each group. The Student’s t test was carried out at a 95% confidence level (*p* < 0.05) and at a 99% confidence level (*p* < 0.01) to separate means that were statistically different.

Selected data were subjected to principal component analysis (PCA) to evaluate the impact of IQOS aerosol exposure on rat liver. Statistical analysis of the data was carried out by using XLSTAT (Addinsoft, New York, NY, USA), 2018 version.

## Data Availability

All data are available in the main text and in Supplementary Material.

## Supplementary Materials

The following supporting information can be downloaded at: https://www.mdpi.com/article/10.3390/ijms241512462/s1. References [50,51,52,53,54,55,56,57,58,59,60,61,62,63,64,65,66,67,68,69,70,71,72] are cited in the Supplementary Materials.

## Abbreviations

CC: conventional cigarettes; GSH, glutathione; CYP, cytochrome P450; Nrf2, nuclear transcription factor 2; WHO, world health organization; NDS, nicotine delivery systems; ENDS, ELECTRONIC nicotine delivery systems; E-cig, electronic cigarettes; FDA, food and drug administration; MRTP, modified-risk tobacco product; PAH, polycyclic aromatic hydrocarbons; ALT, alanine aminotransferase; ROS, reactive oxygen species; DCFH-DA, dichlorodihydrofluorescein diacetate; CP, carbonylated proteins; MDA, malondialdehyde; DCF, dichlorofluorescein; XO, xanthine oxidase; GSSG-red, glutathione disulphide reductase; GSH-Px, GSH peroxidase; SOD, superoxide dismutase; PROD, pentoxyresorufin O-dealkylase; EROD, ethoxyresorufin O-deethylase MROD, methoxyresorufin O-demethylase; pNPH, p-nitrophenol hydroxylase; ECOD, ethoxycoumarin O-deethylase; APND, aminopyrine N-demethylase; GST, glutathione S-transferase, UDPGT, uridine diphosphate-glucuronosyltransferases; FCCP, carbonyl cyanide 4(trifluoromethoxy)phenylhydrazone CS, citrate synthase; TAG, triacylglycerol, STE, free sterol; MAG, monoacylglycerol; FFA, free fatty acids; DAG, diacylglycerols; E-STE, esterified sterols; FA, fatty acids, SFA, saturated fatty acids, PUFA, polyunsaturated fatty acids, MUFA, monounsaturated fatty acids; AI, atherogenic index; TI, thrombogenic index; 24-HC, 24-hydroxycholesterol; 7α-HC, 7α-hydroxycholesterol; 7β-HC, 7β-hydroxycholesterol; 7-KC, 7-ketocholesterol; COR%, cholesterol oxidation ratio; PCA, principal component analysis; IARC, international agency for research on cancer; NAFLD, non-alcoholic fatty liver disease; FRAP, ferric reducing antioxidant power; PSC, sclerosing cholangitis; NASH, nonalcoholic steatohepatitis; TBARs, thiobarbituric acid reactive substance; COX, Cyclooxygenase.
