# Supplementary material for: Potential Harm of IQOS Smoke to Rat Liver
Source: Int J Mol Sci. 2023 Aug 5;24(15):12462. doi: 10.3390/ijms241512462 (PMC10419033; doi:10.3390/ijms241512462)
Supplement: Supplementary file 1 [file ijms-24-12462-s001.zip › ijms-2482284-supplementary.pdf]

Supplementary materials

Potential Harm of IQOS Smoke to Rat Liver

Silvia Granata [1,2,†], Donatella Canistro [1,†], Fabio Vivarelli [1,*], Camilla Morosini [1], Laura Rullo [1], Dario Mercatante [3], Maria Teresa Rodriguez-Estrada [3,4], Alessandra Baracca [5], Gianluca Sgarbi [5], Giancarlo Solaini [5], Severino Ghini [1], Ivan Fagiolino [6], Stefano Sangiorgi [1] and Moreno Paolini [1]

[1] Department of Pharmacy and Biotechnology, Alma Mater Studiorum, University of Bologna, Via Irnerio 48, 40126 Bologna, Italy; silvia.granata3@unibo.it (S.G.); donatella.canistro@unibo.it (D.C.); camilla.morosini2@unibo.it (C.M.); laura.rullo3@unibo.it (L.R.); severino.ghini@unibo.it (S.G.); stefano.sangiorgi9@unibo.it (S.S.); moreno.paolini@unibo.it (M.P.)

[2] Department of Medicine and Surgery, University of Milan–Bicocca, Via Cadore 48, 20900 Monza, Italy

[3] Department of Agricultural and Food Sciences, Alma Mater Studiorum, University of Bologna, Viale Giuseppe Fanin, 40-50, 40127 Bologna, Italy; dario.mercatante2@unibo.it (D.M.); maria.rodriguez@unibo.it (M.T.R.-E.)

[4] Inter-Departmental Centre for Agri-Food Industrial Research, Alma Mater Studiorum, University of Bologna, Via Quinto Bucci 336, 47521 Cesena, Italy

[5] Laboratory of Biochemistry and Mitochondrial Pathophysiology, Department of Biomedical and Neuromotor Sciences, University of Bologna, Via Irnerio, 48, 40126 Bologna, Italy; alessandra.baracca@unibo.it (A.B.); gianluca.sgarbi@unibo.it (G.S.); giancarlo.solaini@unibo.it (G.S.)

[6] Gruppo CSA—S.p.A., Via al Torrente 22, 47923 Rimini, Italy; ifagiolino@csaricerche.com

* Correspondence: fabio.vivarelli3@unibo.it

† These authors contributed equally to this work.

Tissue collection and sub-cellular fraction isolation

Rats were sacrificed following the Italian Ministerial guidelines for the species: they were previously anesthetized with Zoletil 100 (100 mg/kg b.w.). Liver was rapidly excised, washed with PBS pH 7.4 to remove the blood, then snap frozen in liquid nitrogen and stored at –80 °C; tissue homogenate and S9, cytosol and microsomal fractions were obtained as previously described [38]. All of the fractions were frozen in dry ice and then stored at –80 °C. Liver mitochondria were isolated from the liver as follows: tissue was homogenized with a Potter–Elvehjem grinding chamber, pestled in buffer solution (0.22 M mannitol, 0.07 M sucrose, 0.1 mM EGTA, 1 mM EDTA and 20 mM HEPES, pH 7.4, containing 0.4% albumin), and then centrifuged at 2000 rpm for 10 min to remove nuclei and plasma membrane fragments [39]. Supernatants were filtered through a gauge, and the eluted fractions were then centrifuged at 8000 rpm for 10 min. Pellets obtained were washed once in the above buffer devoid of albumin. Finally, mitochondria were resuspended in a buffer containing 0.25 M sucrose, 20 mM HEPES, 0.1 mM EGTA, and 1 mM EDTA, pH 7.4, and promptly assayed. Mitochondrial protein determination was performed by the biuret method [S1], as previously reported [S2].

Respiration rate measurement

Mitochondria were diluted to an optimal concentration with a buffer containing 0.25 M sucrose, 20mM Tris/Cl, 4 mM $MgSO_4$, 0.5 mM EDTA, 10 mM KH_2PO_4, pH 7.4. Maximal oxygen consumption rate, assayed under uncoupling conditions, was measured by adding to mitochondria (0.1 mg) 20 mM succinate (plus 1 mM rotenone) as substrate, and 100 nM carbonyl cyanide 4-(trifluoromethoxy)phenylhydrazone (FCCP) as uncoupler [S3]. The initial rate of respiration was calculated by evaluating the oxygen concentration decline during the first 2 min of reaction. Protein concentration was measured by the Lowry method [40] using bovine serum albumin as standard. The oxygen consumption rate was expressed in nmol/(min x mg of protein) [S4,S5].

Antioxidants

Total Glutathione (GSH) assay. The total amount of GSH was evaluated as follows: TCA 10% was added to the cytosolic fraction and centrifuged at 4000 rpm for 15 min at 4 °C. The supernatant was then treated with Ellman reagent, and readings were performed at λ=412 nm. For the calibration curve, the following concentrations of GSH were used, each treated as previously described: 1.6 mM, 1 mM, 0.75 mM, 0.5 mM, 0.2 mM, 0.02 mM, 2 μM, 0.2 μM, 0.1 Mm [S6,S7].

GSH peroxidase (GSH Px). The incubation mixture for measuring the GSH-px activity contained 50 mM potassium phosphate, 1 mM EDTA, cytosol sample, 10 mM GSH, GSSG reductase (2.4 U/mL) and 1.5 mM NADPH. After incubation at 37 °C for 5 min and addition of tert-butylhydroperoxide, the NADPH consumption was followed at 340 nm for 5 min at 37 °C. GSH peroxidase activity was calculated by use of an extinction coefficient of 6.22 mM^{-1} cm^{-1}, and expressed as nmol of NADPH consumed per minute per milligram of protein from cytosol [S8].

Glutathione disulphide reductase (GSSG Red). GSSG-red activity was measured by adding 1.5 mM NADPH into an assay cuvette containing 50 mM potassium-phosphate buffer, 1 mM EDTA, cytosol sample, and 20 mM GSSG. The generation of NADP+ from NADPH, during the reduction of GSSG, was recorded at 340 nm for 5 min at 37 °C. The GSSG reductase activity was calculated with the extinction coefficient of 6.22 mM^{-1} cm^{-1} and expressed as μmol of NADPH consumed per minute per milligram of protein from cytosol [S9].

DT-Diaphorase. Phosphate buffer 33 mM pH=7.4, 2,6-dichlorophenolindophenol (DCPIP) and the cytosolic fraction were mixed, and the reaction was activated by the cofactor NADPH. The activity was assayed spectrophotometrically at 600 nm by monitoring the reduction of the DCPIP to its colorless form, with NADPH as hydrogen donor. The assay mixture contained 50 mM Tris–HCI (pH 7.5), 1 mM NADPH, and 40 μM DCPIP. The enzymatic activity was calculated with the extinction coefficient of DCPIP (22.1 mM^{-1} cm^{-1}) and expressed as nmol of DCPIP reduced per minute per milligram of protein of cytosolic fraction [S10].
Catalase. The decomposition of the substrate H_2O_2 was measured and followed at λ=240 nm, and CAT activity was expressed as μmol of H_2O_2 consumed per minute per milligram of protein of cytosolic fraction, using a molar extinction coefficient of 43.6 M^{-1} cm^{-1} [45].
Superoxide dismutase (SOD). Briefly, the activity was assayed in cytosol fraction spectrophotometrically at 320 nm by monitoring the generation of adenochrome, one of the main products of epinephrine autoxidation at pH 10.2. SOD was calculated by using the extinction coefficient of 4.02 per mM^{-1} cm^{-1} and expressed as moles of epinephrine oxidized/min per mg protein, derived by subtracting each test curve from the epinephrine autoxidation standard curve [S12].
Xanthine Oxidase (XO). The activity was determined in the cytosol by quantifying the formation of uric acid spectrophotometrically at 290 nm. The reaction mixture contained 50 mM sodium phosphate buffer (pH 7.8) and hypoxantine (50 μM final concentration), and it was incubated at 37 °C for 5 min. The reaction was started with the addition of NAD+ [S13]

Phase I enzymes

CYP-linked monooxygenases were assessed as previously reported in the microsomal fraction [45]. Briefly, p-Nitrophenol hydroxylase (p-NPH, CYP2E1) was quantified by measuring 4-nitrocatechol formation at λ=546 nm (ε =10.28 mM^{-1} cm^{-1}). The activity was measured in a final volume of 2 mL containing 2 mM p-nitrophenol in 50 mM Tris–HCl buffer (pH 7.4), 5 mM $MgCl_2$ and a NADPH-generating system consisting of 0.4 mM NADP+, 30 mM isocitrate, 0.2 U of isocitrate dehydrogenase and 1.5 mg of proteins. After 10 min at 37 °C, the reaction was stopped by addition of 0.5 mL of 0.6 N perchloric acid. Pentoxyresorufin *O*-dealkylase (PROD, CYP2B1/2), ethoxyresorufin O-deethylase (EROD, CYP 1A1) and methoxyresorufin *O*-demethylase (MROD, CYP1A2) were measured through the quantity of resorufin formation, using pentoxyresorufin, ethoxyresorufin and methoxyresorufin as substrates, respectively (n=8 measurements per group). For measurement of PROD activity, the reaction mixture consisted of 0.025 mM $MgCl_2$, 200 mM pentoxyresorufin, 0.32 mg of proteins and 130 mM NADPH in 2.0 mL 0.05 M Tris–HCl buffer (pH 7.4). Resorufin formation at 37 °C was calculated by comparing the rate of increase in relative fluorescence to the fluorescence of known amounts of resorufin (excitation 562 nm, emission 586 nm). EROD and MROD activities were measured in the same way, except that the substrates were 1.7 mM ethoxyresorufin and 5 mM methoxyresorufin, respectively. Ethoxycoumarin *O*-deethylase (ECOD, CYP1A1, 1A2 and 2B) was determined following umbelliferone production with EX=390 nm, EM=440 nm and slit=5/5 [S14]. The 2.6 mL incubation mixture consisted of 1 mM ethoxycoumarin, 5 mM $MgCl_2$, an NADPH-generating system and 25 μL of sample. After 5 min of incubation at 37 °C, the reaction was stopped with 85 μL of 0.31 M TCA. The pH of the mixture was adjusted to about 10 by adding 0.65 mL of 1.6 M NaOH–glycine buffer (pH 10.3). The aminopyrine N-demethylase (APND, CYP3A1/2) was assessed by quantifying CH_2O release, reading at 412 nm [S15,S16].

Western blot

Proteins were denatured by incubating the mix at 75 °C for 15 min, followed by ice-shocking and loading into the wells for the electrophoresis run. Each group had 50 µg of proteins in a volume of 40 µL per well loaded (CTR=4 wells, IQOS=5 wells); in the first well, 6 µL of SeaBlue Plus pre-stained protein standard was placed. Electrophoresis was run at 80 V for 120 min, in running buffer 1x. At the end, the gel was inserted in the transfer box; the procedure was conducted in distilled water and transfer buffer 1x, for 120 min at 10 V. Afterwards, the nitrocellulose membrane was withdrawn, washed, blocked with milk 5% for 3 h, and was then ready to be treated. After the blocking, the membrane was incubated with the primary antibody overnight; the day after, the antibody was retrieved, and the membrane was washed with TBStween 1x two times for 10 min and two times for 5 min; then, it was incubated for 3 h with the secondary antibody and washed as before. Afterwards, the membrane was incubated with ECL (Clarity Western ECL substrate Bio- Rad) for 5 min and then read at the ChemiDoc. If the antibody previously used was the phospho one, then the membrane was blocked with milk 5% for 1.5 h and treated with the total antibody as previously described. NrF2 with antibody anti-NrF2 (Nrf2 Polyclonal Antibody, Thermo Fischer, Item no. PA5-68817; 1:500), p38 and p-p38 with antibodies anti-p38 (rabbit polyclonal antibody to p38 MAPK; ABclonal 1:900) and anti-p-p38 (rabbit polyclonal antibody to phospho-p38 MAPK; ABclonal 1:1000) were investigated, and α-tubulin was used as normalizing factor, with the antibody anti-tubulin goat (eBioscience™ Anti- α Tubulin, Invitrogen, Item no. 4,348,184; 1:2000). As secondary antibodies, an anti-mouse IgG (Gt anti-Ms IgG Secondary Antibody, Invitrogen, Item no. TG267017) for the tubulin and an anti-rabbit IgG (Gt anti-Rb IgG Secondary Antibody, Invitrogen, Item no. TG266717) for the others were used, both at 1:2000. All of the proteins investigated were tested in two different membranes.

Lipid extraction

Lipids were extracted in rat liver according to a modified version of the Folch method [49]. A lipid fraction of 4 g of liver added with 0.927 mg of 5α-cholestane (internal standard for the quantification of main lipid classes; Sigma Chemical, St. Louis, USA) was extracted using a chloroform:methanol solution (1:1, v/v) followed by the addition of another aliquot of chloroform. After mixing with 1 M KCl, the organic phase was separated and taken to dryness; the fat content was determined gravimetrically; eight independent replicates for each animal group were carried out.

Lipid profile

The profile of the main lipid classes was determined as follows: starting from the extract obtained with the modified Folch method, an aliquot of the solution equal to 20 mg of fat was taken, added with 1 mL of *n*-hexane and injected into a gas chromatograph coupled to a flame ionization detector (GC-FID); the instrument was a Shimadzu QP-2,010 (Kyoto, Japan) interfaced with a computerized system for data acquisition (GC solution ver. 2.5, Shimadzu, Japan). An SE-52 fused silica column (10 m x 0.32 mm x 0.1 µm film thickness) coated with 5% phenyl and 95% methyl-polysiloxane was used. Oven temperature was programmed from 100 °C to 355 °C at a rate of 5 °C/min kept for 20 min. Injector and detector temperatures were both set at 355 °C. Helium was used as carrier gas at a flow rate of 2.02 mL/min and a pressure of 32.2 kPa. The split ratio was 1:25. Different lipid classes were identified using diverse commercial standards (Sigma-Aldrich Chemical Company, St. Louis, MO, USA). The amount of each lipid class was quantified using the internal standard method with

the response factor of each main lipid class (estimated using suitable commercial standards). Eight replicates were analyzed for each animal group [S17,S18].

Total fatty acid composition in liver lipid matter

About 20 mg of liver lipid matter was treated with 200 μL of diazomethane in order to esterify free fatty acids (FFA) [S19]. Tridecanoic methyl ester (40 μL of a solution 3.1822 mg mL^{-1} of C13:0; internal standard) was then added, and the mixture was transesterified with 40 μL of 2 N KOH in methanol [S20] (European Commission, 2002), vortexed for 1 min, left standing for 5 min, and centrifuged at 1500 *g* for 3 min. Supernatant was placed in a vial and then analyzed by GC-FID [S21]. The GC-FID instrument was a GC8000 series (Fisons Instruments, Milan, Italy) interfaced with a computerized system for data acquisition (Chromcard Data System, ver. 2.3.1, Fisons Instruments). An RTX 2330 fused-silica column (30 m × 0.25 mm × 0.2 μm film thickness) (Restek, USA) coated with 90% biscyanopropyl and 10% cyanopropyl-phenyl polysiloxane was used. Oven temperature was programmed from 100 °C to 240 °C at a rate of 5 °C min^{-1}, and the final temperature was kept for 20 min. Injector and detector temperatures were both set at 250 °C. Helium was used as carrier gas at a constant pressure of 260 kPa. The split ratio was 1:30. Each fatty acid (FA) was identified by comparing its retention time with that of a commercial FA methyl ester standard solution (NU-CHEK 463 Mix, NU-CHEK PREP Inc., USA). The GC response factor of each FA was calculated by using the NU-CHEK 463 standard mixture and the internal standard (C13:0). Quantification of FAME was carried out according to the internal standard method. Eight independent replicates for each animal group were analyzed.

The Δ-desaturase index was calculated as follows: [(C20:2n-6 + C20:4n-6 + C20:5n-3 + C22:5n-3 + C22:6n-3)/(C18:2n-6 + C18:3n-3+ C20:2n-6 + C20:4n-6 + C20:5n-3 + C22:5n-3 + C22:6n-3) X 100]. Based on the total FA composition, the atherogenic index (AI) and thrombogenic index (TI) were also determined [S22] according to the following equations:

AI= (C12:0 + 4 × C14:0 + C16:0)/(ΣMUFA + ΣPUFA n-6 + ΣPUFA n-3);

TI= (C14:0 + C16:0 + C18:0)/[(0.5 × ΣMUFA) + (0.5 × ΣPUFA n-6) + (3 × ΣPUFA n-3) + (ΣPUFA n-3/ΣPUFA n-6)].

Cholesterol and cholesterol oxysterol products (COPs)

Lipid extract containing internal standards (41.64 µg of betulinol (Sigma Chemical, St. Louis, USA) and 5.35 µg of 19-hydroxycholesterol (Steraloids, Newport, Rhode Island, USA) for cholesterol and COPs, respectively) was subjected to cold saponification. One-tenth of the unsaponifiable matter was used to determine the sterol composition, while the remaining nine-tenths were purified by SPE-NH_2 for COP quantification. Both cholesterol and COPs fractions were silylated at 40 °C for 20 min, taken to dryness under a nitrogen stream, re-dissolved in *n*-hexane and injected in Fast GC/MS. Mass spectra were acquired in full scan mode (total ion current (TIC)), while they were integrated with single ion monitoring (SIM) mode using the characteristic ions with a high abundance. Identification of cholesterol and COPs was carried out by comparing their mass spectra and retention times with those of the corresponding chemical standards (Sigma Chemical, Steraloids (Newport, Rhode Island, USA) and Avanti Polar Lipids (Alabaster, Alabama, USA)); their quantification was carried out by means of calibration curves built for each compound. Cholesterol and COPs were expressed as mg g^{-1} and

µg g^{-1} of liver, respectively; eight independent replicates for each group were carried out; the rate of total cholesterol oxidation (%OR) was also determined according to the following formula: %OR = (Total COPs/Total cholesterol) x 100 [S23].

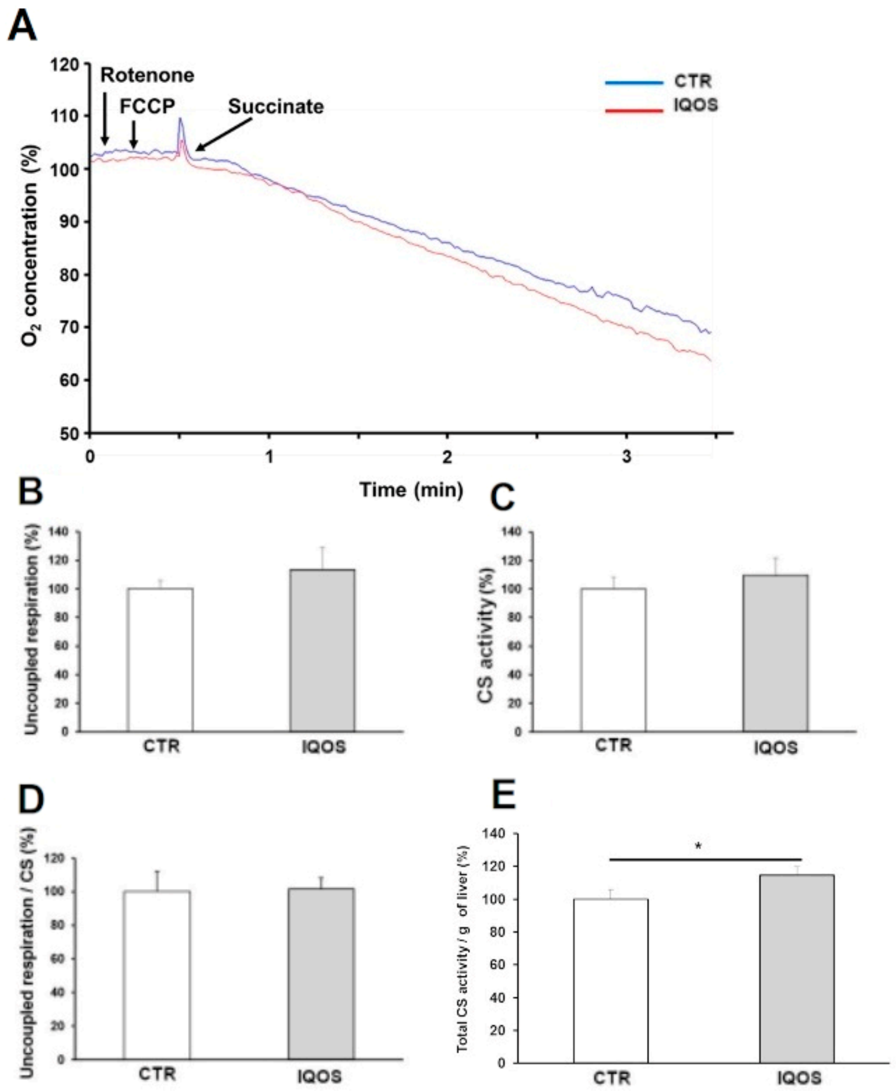

Figure S1*. *IQOS smoking's effect on liver mitochondria. *(a) Typical Complex II-driven oxygen consumption traces of mitochondria isolated from liver of control (blue line) and IQOS-treated (red line) rats. Uncoupled respiration obtained in the presence of saturating concentration of both succinate and FCCP is shown. The saturating oxygen concentration (100%) at 37 °C is 204.1 µM. Due to the technical characteristics of the oxygen electrode, the O_2 concentration at the start of the assay might slightly differ from 100%. b-d) Mitochondria were isolated from control and IQOS-treated rats. (b) Complex II-sustained uncoupled respiration was assessed in mitochondria in the presence of FCCP. Data are expressed as % of controls (mean value: 47.13 ± 2.69 nmol min^{-1} mg^{-1} of protein). (c) CS activity, considered a marker of mitochondrial content (Costanzini et al. 2019), was measured as reported in section 2. Data are expressed as % of controls (the mean value of CS activity was: 114.72 ± 18.98 nmol min^{-1} mg^{-1} of protein). (d) Oxygen consumption rate normalized to CS. Data are expressed as % of controls (mean value: 0.421 ± 0.071). Each sample was assayed at least in triplicate. (e) Total CS activity present in the crude homogenate normalized to the gram of wet liver weighted just prior to mitochondria*

*isolation. Data are expressed as % of controls (mean value: 12.43 ± 1.44 nmol min^{-1} mg^{-1} g^{-1} of liver). * p< 0.05 indicates the statistical significance of data.*

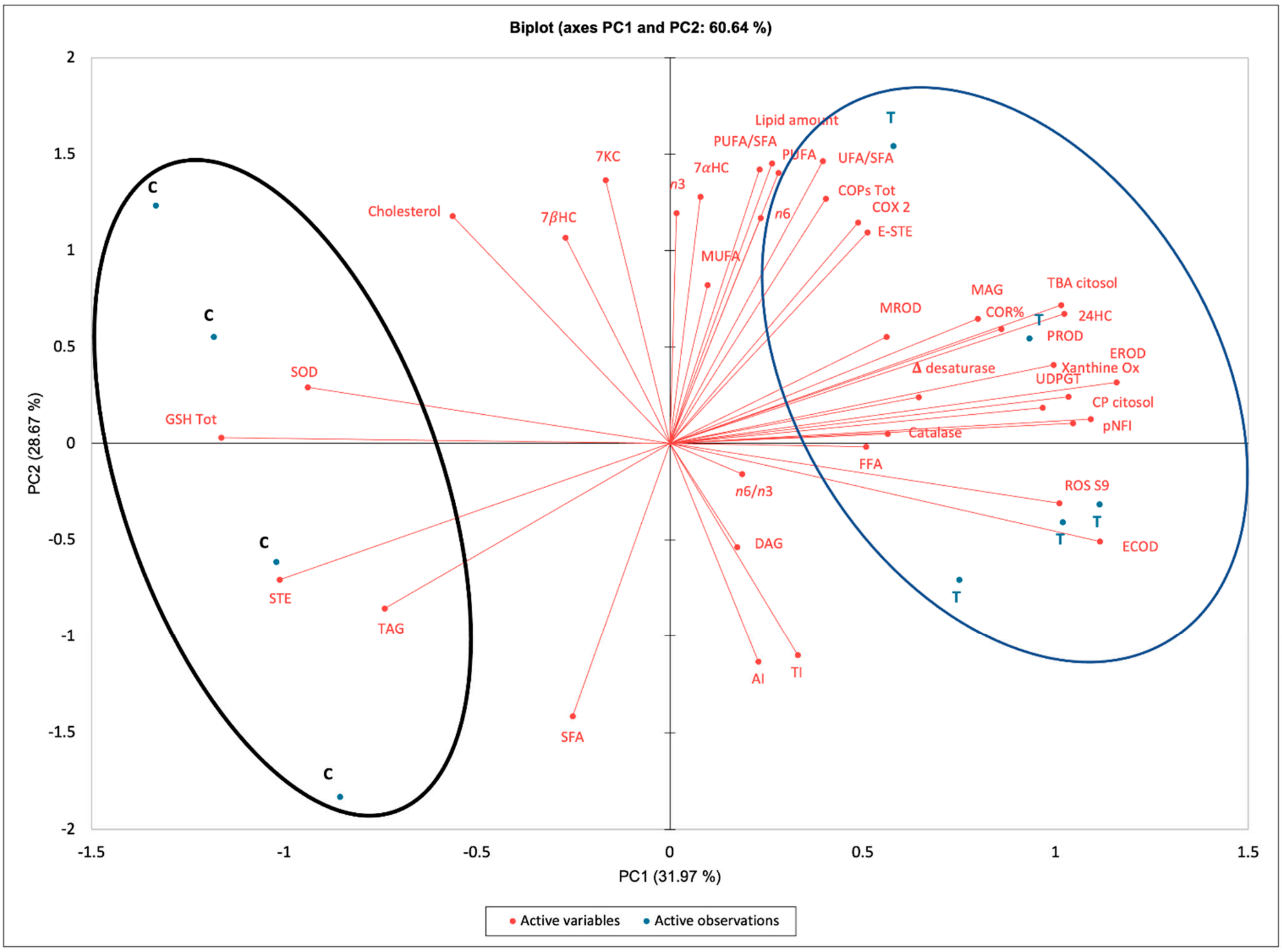

Figure S2. Biplot of selected data and treatment. *7α-HC, 7α-hydroxycholesterol; 7β-HC, 7β-hydroxycholesterol; 7-KC, 7-ketocholesterol; 24-HC, 24-hydroxycholesterol; AI, atherogenic index; COX 2, cyclooxygenase 2; COPs; cholesterol oxidation products; COR%, cholesterol oxidation ratio; CP, carbonylated proteins; DAG, diacylglycerols; ECOD, ethoxycoumarin O-deethylase; EROD, ethoxyresorufin O-deethylase; E-STE, esterified sterols; FFA, free fatty acids; GSH tot, glutathione; MAG, monoacylglycerols; MROD, methoxyresorufin O-demethylase; MUFA, monounsaturated fatty acids; pNFI, p-nitrophenol hydroxylase; PROD, pentoxyresorufin O-dealkylase; PUFA, polyunsaturated fatty acids; ROS S9, reactive oxygen species; SFA, saturated fatty acids; SOD, superoxide dismutase; STE, free sterols; TAG, triacylglycerols; TBA, thiobarbituric acid reactive substances; TI, thrombogenic index; UDPGT, UDP-glucuronosyl transferase; UFA, unsaturated fatty acids.*

Table S1. Chemical characterization of IQOS mainstream.

Parameter	IQOS (µg/HEETS stick)	Method
**Dusts - inhalable fraction**	100 ± 7	M.U. 1998:13
**Nitrogen oxides (as NO_2)**	< 0.4	NIOSH 6014 1994
**Nicotine**	113 ± 26	NIOSH 2551 1998
**ALDEHYDES**		-
**Formaldehyde**	3.00 ± 0.45	EPA 8315A 1996
**Acetaldehyde**	14.0 ± 2.1	EPA 8315A 1996
**Propionaldehyde**	1.00 ± 0.15	EPA 8315A 1996
**Butyraldehyde (Butanal)**	1.00 ± 0.15	EPA 8315A 1996
**Crotonaldehyde (2-butenal)**	< 1	EPA 8315A 1996
**Acrolein (2-Propenal)**	< 1	EPA 8315A 1996
**Phenols and Cresols**	< 1	NIOSH 2546 1994
**Volatile Organic Compounds (VOCs)**	1.09 ± 0.27	UNI EN ISO 16017-1:2002
**Benzene**	0.078 ± 0.020	UNI EN ISO 16017-1:2002
**Ethylbenzene**	0.078 ± 0.020	UNI EN ISO 16017-1:2002
**Toluene**	0.183 ± 0.046	UNI EN ISO 16017-1:2002
**Xylenes**	0.56 ± 0.14	UNI EN ISO 16017-1:2002
**Alkylbenzenes**	0.189 ± 0.047	UNI EN ISO 16017-1:2002
**Metals in the particulate fraction**		-
**Arsenic**	< 50	UNI EN 14902:2005/EC1:2008 + UNI EN ISO 17294-2:2016
**Cadmium**	< 50	UNI EN 14902:2005/EC1:2008 + UNI EN ISO 17294-2:2016
**Cobalt**	< 50	UNI EN 14902:2005/EC1:2008 + UNI EN ISO 17294-2:2016
**Chrome**	< 200	UNI EN 14902:2005/EC1:2008 + UNI EN ISO 17294-2:2016
**Iron**	< 500	UNI EN 14902:2005/EC1:2008 + UNI EN ISO 17294-2:2016
**Manganese**	< 50	UNI EN 14902:2005/EC1:2008 + UNI EN ISO 17294-2:2016
**Nickel**	< 200	UNI EN 14902:2005/EC1:2008 + UNI EN ISO 17294-2:2016
**Lead**	< 50	UNI EN 14902:2005/EC1:2008 + UNI EN ISO 17294-2:2016
**Copper**	350 ± 53	UNI EN 14902:2005/EC1:2008 + UNI EN ISO 17294-2:2016
**Thallium**	< 1000	UNI EN 14902:2005/EC1:2008 + UNI EN ISO 17294-2:2016
**Tellurium**	< 200	UNI EN 14902:2005/EC1:2008 + UNI EN ISO 17294-2:2016
**Vanadium**	< 50	UNI EN 14902:2005/EC1:2008 + UNI EN ISO 17294-2:2016
**Zinc**	< 500	UNI EN 14902:2005/EC1:2008 + UNI EN ISO 17294-2:2016
**Mercury**	< 100	UNI EN 14902:2005/EC1:2008 + UNI EN ISO 17294-2:2016
**PAHs**		-
**Naphthalene**	<0.1	DM 05/05/2015 GU n°128 05/06/2016
**Acenaphthylene**	<0.1	DM 05/05/2015 GU n°128 05/06/2017
**Acenaphthene**	<0.1	DM 05/05/2015 GU n°128 05/06/2018
**Fluorene**	<0.1	DM 05/05/2015 GU n°128 05/06/2019
**Phenanthrene**	2.4 ± 1.0	DM 05/05/2015 GU n°128 05/06/2020
**Anthracene**	<0.1	DM 05/05/2015 GU n°128 05/06/2021
**Fluoranthene**	6.2 ± 2.7	DM 05/05/2015 GU n°128 05/06/2022
**Pyrenees**	4.4 ± 1.9	DM 05/05/2015 GU n°128 05/06/2023
**Benzo(a)anthracene**	1.7 ± 0.7	DM 05/05/2015 GU n°128 05/06/2024
**Cyclopenta(c,d)pyrene**	0.4 ± 0.2	DM 05/05/2015 GU n°128 05/06/2025

**Chrysene**	1.8 ± 0.8	DM 05/05/2015 GU n°128 05/06/2026
**Benzo(b)+(j)fluoranthene**	1.1 ± 0.5	DM 05/05/2015 GU n°128 05/06/2027
**Benzo(k)fluoranthene**	0.4 ± 0.2	DM 05/05/2015 GU n°128 05/06/2028
**Benzo(e)pyrene**	0.7 ± 0.3	DM 05/05/2015 GU n°128 05/06/2029
**Benzo(a)pyrene**	0.4 ± 0.2	DM 05/05/2015 GU n°128 05/06/2030
**Indeno(1,2,3,c,d,)pyrene**	0.3 ± 0.1	DM 05/05/2015 GU n°128 05/06/2031
**Dibenzo(ac)+(ah)anthracene**	<0.1	DM 05/05/2015 GU n°128 05/06/2032
**Benzo(g.h,i)perylene**	0.5 ± 0.2	DM 05/05/2015 GU n°128 05/06/2033
**Dibenzo (a,l) pyrene**	<0.1	DM 05/05/2015 GU n°128 05/06/2034
**Dibenzo(a,e)fluoranthene**	<0.1	DM 05/05/2015 GU n°128 05/06/2035
**Dibenzo (a,e) pyrene**	<0.1	DM 05/05/2015 GU n°128 05/06/2036
**Dibenzo (a,i) pyrene**	<0.1	DM 05/05/2015 GU n°128 05/06/2037
**Dibenzo (a,h) pyrene**	<0.1	DM 05/05/2015 GU n°128 05/06/2038
**Summation of IPA analytical values**	20.3 ± 8.9	DM 05/05/2015 GU n°128 05/06/2039

Table S2. Fatty acid composition (expressed as mg/100 mg of lipids), main fatty acid classes (expressed as % of total fatty acids), main fatty acid ratios, Δ-desaturase, atherogenic and thrombogenic indices of rat livers.

	**Control**	**IQOS**
**C 14:0**	0.39±0.07	0.40±0.12
**C 14:1**	0.24±0.02	0.34±0.10
**C 16:0**	20.57±0.65	22.27±3.14
**C 16:1 *n-7***	2.12±0.31	2.24±0.53
**C 17:0**	0.33±0.05	0.51±0.04
**C 17:1**	0.50±0.16	0.23±0.02
**C 18:0**	11.39±0.83	12.39±0.54
**C 18:1 *n-9***	6.66±0.50	6.89±0.66
**C 18:1 *c11***	6.66±1.57	6.65±0.46
**C 18:2**	15.55±2.09	15.75±0.61
**C 18:3 *n-3***	0.23±0.04	0.18±0.03
**C 20:1**	0.07±0.01	0.09±0.01
**C 20:2**	0.16±0.03	0.67±0.08
**C 20:3**	0.45±0.05	0.11±0.08
**C 22:0**	16.96±1.39	16.91±1.20
**C 20:5**	0.29±0.02	0.29±0.02
**C 24:0**	12.08±4.02	7.88±1.66
**C 22:3**	0.08±0.04	0.21±0.09
**C 22:4**	0.11±0.01	0.19±0.07

**C 22:5**	0.97±0.13	0.93±0.16
**C 22:6**	4.20±0.37	4.73±0.64
**SFA**	61.72±3.35	60.35±1.03
**MUFA**	16.24±1.63	16.59±0.51
**PUFA**	22.04±2.31	23.06±1.04
**PUFA *n-3***	6.14±0.53	6.24±0.75
**PUFA *n-6***	16.79±2.15	17.54±0.62
n-6/n-3	2.74±0.35	2.84±0.36
**PUFA/SFA**	0.36±0.05	0.38±0.02
**UFA/SFA**	0.62±0.09	0.66±0.03
***Δ*-desaturase index**	24.40±2.74	29.29±2.58
**AI**	0.57±0.06	0.59±0.10
**TI**	0.92±0.10	0.98±0.15

Results as reported as means ± s.d. of eight independent replicates. *n.s.* no significant differences between samples (Student t-test $p<0.05$). AI, atherogenic index; MUFA, monounsaturated fatty acids; PUFA, polyunsaturated fatty acids; SFA, saturated fatty acids; TI, thrombogenic index; UFA, unsaturated fatty acids.

References

S1. Gornall, A.G.; Bardawill, C.J.; David, M.M. Determination of serum proteins by means of the biuret reaction. *J. Biol. Chem.* **1949**, *177*, 751–766.

S2. Solaini, G.; Baracca, A.; Gabellieri, E.; Lenaz, G. Modification of the mitochondrial F1-ATPase epsilon subunit, enhancement of the ATPase activity of the IF1-F1 complex and IF1-binding dependence of the conformation of the epsilon subunit. *Biochem. J.* **1997**, *327 Pt 2*, 443–448. https://doi.org/10.1042/bj3270443.

S3. Bosetti, F.; Baracca, A.; Lenaz, G.; Solaini, G. Increased state 4 mitochondrial respiration and swelling in early post-ischemic reperfusion of rat heart. *FEBS Lett.* **2004**, *563*, 161–164. https://doi.org/10.1016/S0014-5793(04)00294-7.

S4. Aleardi, A.M.; Benard, G.; Augereau, O.; Malgat, M.; Talbot, J.C.; Mazat, J.P.; Letellier, T.; Dachary-Prigent, J.; Solaini, G.C.; Rossignol, R. Gradual alteration of mitochondrial structure and function by beta-amyloids: Importance of membrane viscosity changes, energy deprivation, reactive oxygen species production, and cytochrome c release. *J. Bioenerg. Biomembr.* **2005**, *37*, 207–225. https://doi.org/10.1007/s10863-005-6631-3.

S5. Sgarbi, G.; Liuzzi, F.; Baracca, A.; Solaini, G. Resveratrol preserves mitochondrial function in a human post-mitotic cell model. *J. Nutr. Biochem.* **2018**, *62*, 9–17. https://doi.org/10.1016/j.jnutbio.2018.07.017.

S6. Ellman, G.L. Tissue sulfhydryl groups. *Arch. Biochem. Biophys.* **1959**, *82*, 70–77. https://doi.org/10.1016/0003-9861(59)90090-6.

S7. Sedlak, J.; Lindsay, R.H. Estimation of total, protein-bound, and nonprotein sulfhydryl groups in tissue with Ellman's reagent. *Anal. Biochem.* **1968**, *25*, 192–205. https://doi.org/10.1016/0003-2697(68)90092-4.

S8. Melega, S.; Canistro, D.; Pagnotta, E.; Iori, R.; Sapone, A.; Paolini, M. Effect of sprout extract from Tuscan black cabbage on xenobiotic-metabolizing and antioxidant enzymes in rat liver. *Mutat. Res.* **2013**, *751*, 45–51. https://doi.org/10.1016/j.mrgentox.2012.10.013.

S9. Pavan, B.; Dalpiaz, A.; Marani, L.; Beggiato, S.; Ferraro, L.; Canistro, D.; Paolini, M.; Vivarelli, F.; Valerii, M.C.; Comparone, A.; et al. Geraniol Pharmacokinetics, Bioavailability and Its Multiple Effects on the Liver Antioxidant and Xenobiotic-Metabolizing Enzymes. *Front. Pharmacol.* **2018**, *9*, 18. https://doi.org/10.3389/fphar.2018.00018.

S10. Ernster, L. DT diaphorase. In *Methods in Enzymology*; Academic Press: Cambridge, MA, USA, 1967; Volume 10, pp. 309–317. https://doi.org/10.1016/0076-6879(67)10059-1.

S11. Kumar, K.A.; Reddy, T.C.; Reddy, G.V.; Reddy, D.B.; Mahipal, S.V.; Sinha, S.; Gaikwad, A.N.; Reddanna, P. High-throughput screening assays for cyclooxygenase-2 and 5-lipoxygenase, the targets for inflammatory disorders. *Indian J. Biochem. Biophys.* **2011**, *48*, 256–261.

S12. Misra, H.P.; Fridovich, I. The role of superoxide anion in the autoxidation of epinephrine and a simple assay for superoxide dismutase. *J. Biol. Chem.* **1972**, *247*, 3170–3175.

S13. Shintani, H. Determination of Xanthine Oxidase. *Pharm. Anal. Acta* **2013**, *S7*, 004. https://doi.org/10.4172/2153-2435.S7-004.
S14. Aitio, A. A simple and sensitive assay of 7-ethoxycoumarin deethylation. *Anal. Biochem.* **1978**, *85*, 488–491. https://doi.org/10.1016/0003-2697(78)90245-2.
S15. Mazel, P. Experiments illustrating drug metabolism in vitro. In *Fundamentals of Drug Metabolism and Drug Disposition*; Williams & wilkins: Philadelphia, PA, USA, 1971; pp. 546-582.
S16. Nash, T. The colorimetric estimation of formaldehyde by means of the Hantzsch reaction. *Biochem. J.* **1953**, *55*, 416–421. https://doi.org/10.1042/bj0550416.
S17. Toschi, T.G.; Cardenia, V.; Bonaga, G.; Mandrioli, M.; Rodriguez-Estrada, M.T. Coffee silverskin: Characterization, possible uses, and safety aspects. *J. Agric. Food Chem.* **2014**, *62*, 10836–10844. https://doi.org/10.1021/jf503200z.
S18. Luise, D.; Cardenia, V.; Zappaterra, M.; Motta, V.; Bosi, P.; Rodriguez-Estrada, M.T.; Trevisi, P. Evaluation of Breed and Parity Order Effects on the Lipid Composition of Porcine Colostrum. *J. Agric. Food Chem.* **2018**, *66*, 12911–12920. https://doi.org/10.1021/acs.jafc.8b03097.
S19. Fieser, L.F.; Fieser, M. *Reagents for Organic Chemistry*; Wiley: New York, NY, USA, 1967; pp. 191–192.
S20. European Commision (EC). Allegate X. B. Regulation 796/02. *Off. J. Eur. Communities* **2002**, *L128*, 14–18.
S21. Cardenia, V.; Rodriguez-Estrada, M.T.; Baldacci, E.; Lercker, G. Health-related lipids components of sardine muscle as affected by photooxidation. *Food Chem. Toxicol.* **2013**, *57*, 32–38. https://doi.org/10.1016/j.fct.2013.02.053.
S22. Ulbricht, T.L.; Southgate, D.A. Coronary heart disease: Seven dietary factors. *Lancet* **1991**, *338*, 985–992. https://doi.org/10.1016/0140-6736(91)91846-m.
S23. Cardenia, V.; Massimini, M.; Poerio, A.; Venturini, M.C.; Rodriguez-Estrada, M.T.; Vecchia, P.; Lercker, G. Effect of dietary supplementation on lipid photooxidation in beef meat, during storage under commercial retail conditions. *Meat Sci.* **2015**, *105*, 126–135. https://doi.org/10.1016/j.meatsci.2015.02.010.
